# Tracking Radiolabeled Endothelial Microvesicles Predicts Their Therapeutic Efficacy: A Proof-of-Concept Study in Peripheral Ischemia Mouse Model Using SPECT/CT Imaging

**DOI:** 10.3390/pharmaceutics14010121

**Published:** 2022-01-04

**Authors:** Romain Giraud, Anaïs Moyon, Stéphanie Simoncini, Anne-Claire Duchez, Vincent Nail, Corinne Chareyre, Ahlem Bouhlel, Laure Balasse, Samantha Fernandez, Loris Vallier, Guillaume Hache, Florence Sabatier, Françoise Dignat-George, Romaric Lacroix, Benjamin Guillet, Philippe Garrigue

**Affiliations:** 1 C2VN, INSERM, INRAE, Aix Marseille University, 13385 Marseille, France; giraud.romain.amu@gmail.com (R.G.); anais.moyon@ap-hm.fr (A.M.); stephanie.simoncini@univ-amu.fr (S.S.); anne-claire.duchez@univ-amu.fr (A.-C.D.); corinne.chareyre@univ-amu.fr (C.C.); ahlem.bouhlel@univ-amu.fr (A.B.); laure.balasse@univ-amu.fr (L.B.); loris.vallier@gmail.com (L.V.); guillaume.hache@univ-amu.fr (G.H.); florence.sabatier-malaterre@univ-amu.fr (F.S.); francoise.dignat-george@univ-amu.fr (F.D.-G.); romaric.lacroix@univ-amu.fr (R.L.); benjamin.guillet@univ-amu.fr (B.G.); 2 CERIMED, CNRS, Marseille, Aix Marseille University, 13385 Marseille, France; vincent.nail@univ-amu.fr (V.N.); samantha.fernandez@univ-amu.fr (S.F.); 3 Radiopharmacy, Pôle Pharmacie, University Hospitals of Marseille, APHM, 13005 Marseille, France; 4 Department of Hematology and Vascular Biology, University Hospitals of Marseille, APHM, 13005 Marseille, France

**Keywords:** microvesicles, ischemia, nuclear imaging, theranostics, cell-free therapy, angiogenesis

## Abstract

Microvesicles, so-called endothelial large extracellular vesicles (LEVs), are of great interest as biological markers and cell-free biotherapies in cardiovascular and oncologic diseases. However, their therapeutic perspectives remain limited due to the lack of reliable data regarding their systemic biodistribution after intravenous administration. Methods: Applied to a mouse model of peripheral ischemia, radiolabeled endothelial LEVs were tracked and their in vivo whole-body distribution was quantified by microSPECT/CT imaging. Hindlimb perfusion was followed by LASER Doppler and motility impairment function was evaluated up to day 28 post-ischemia. Results: Early and specific homing of LEVs to ischemic hind limbs was quantified on the day of ischemia and positively correlated with reperfusion intensity at a later stage on day 28 after ischemia, associated with an improved motility function. Conclusions: This concept is a major asset for investigating the biodistribution of LEVs issued from other cell types, including cancer, thus partly contributing to better knowledge and understanding of their fate after injection.

## 1. Introduction

Critical limb ischemia (CLI) is an advanced form of peripheral artery disease. CLI has a growing incidence, from 500 to 1000 new cases per million every year, in Western Europe and North America [1]. Despite progresses in public health, hygiene, cardiovascular events prevention, or drugs and medical devices developments, CLI remains associated with a decreased quality of life and a high morbidity and mortality worldwide, being responsible for a high rate of amputation [2]. Vascular regenerative medicine has a key role to play in amputation prevention: cell therapies based on endothelial progenitors or mesenchymal stem cells display interesting properties towards ischemic diseases, mainly granted for factor release, immunomodulation, and inflammatory capacity, among other properties [3]. However, many clinical trials are necessary before achieving a specific, safe and effective cell-based therapy with reproducible and standardized production for patients with no other therapeutic alternatives [4].

Dozens of studies and clinical trials are currently evaluating extracellular vesicles as biomarkers in diseases related with vascular dysfunction or cancer [5,6,7,8]. Large extracellular vesicles (LEVs, formerly named “microvesicles”) are cell-derived submicron vesicles, issued from plasma membrane bubbling. LEVs are secreted and sport a high payload for proteins, RNA, and other substances carried to target cells [9,10]. Involved in numerous pathophysiological processes, endothelial LEVs have gained a considerable growing interest in the literature, not only as biological markers for diagnostic and prognostic purposes, but also as biotherapeutic agents in cardiovascular, neuronal, and oncologic diseases [11,12]. Their characterization, isolation, and purification guidelines have been recently established [13].

Over the past decade, the therapeutic potential of endothelial LEVs as cell-free biotherapy for ischemic diseases emerged from the literature [14,15,16]. Still, clinical therapeutic LEV developments remain challenging as many technical and scientific limitations have been reported, among which is their largely unknown biodistribution after systemic administration [4]. Recent works in the literature mainly focused on the in vivo biodistribution of small extracellular vesicles (SEVs, formerly named “exosomes”) originating from non-endothelial cells, such as tumor cells or progenitor cells [17,18,19,20]. Not only do LEVs and SEVs display differences in their size and their qualitative and quantitative compositions of proteins, lipids, nucleic acids, and surface antigens, but most importantly, endothelial-derived LEV biodistribution should not be blindly extrapolated from biodistribution data of non-endothelial extracellular vesicles. 

Furthermore, clinical applications of endothelial LEVs for therapeutic purposes need not only standardization of preparation and measurement for reproducibility and safety, but also solid data regarding their biodistribution and homing after systemic administration for safety, mechanistic, and optimization concerns [21].

The objective of this work was therefore to overcome the absence of data regarding endothelial LEV biodistribution. Radionuclide imaging was considered for tracking LEVs, as already used in clinical practices to track erythrocytes, platelets, and neutrophils, and offering the best sensitivity for detecting and quantifying nano- to picomolar-range molecular processes in vivo [22,23]. SPECT/CT imaging modality had already been used for tracking other types of extracellular vesicles, such as SEVs, from diverse cellular origins [18]. To radiolabel LEVs, we chose to use [^99m^Tc]Tc-Annexin-V-128, which binds to phosphatidylserine molecules present in the LEV membrane and has previously been used to radiolabel EVs considering that the bind between [^99m^Tc]Tc-AnnV and phosphatidylserines was strong and stable at physiological calcium concentration in the serum [24,25].

Once radiolabeled, the in vivo biodistribution of LEVs was quantified using microSPECT/CT imaging in a preclinical model of hind limb ischemia, and the one-month therapeutic outcome was evaluated through the hind limb revascularization follow-up and the motricity.

## 2. Materials and Methods

### 2.1. Production of Endothelial LEVs

Methods for LEV production and purification followed the latest recommendations from the International Society for Extracellular Vesicles [13] and were submitted to the Transparent Reporting and Centralizing Knowledge in Extracellular Vesicle research consortium (EV-TRACK ID: EV200112) [26,27].

Human umbilical vein endothelial cells (HUVECs, courtesy from the Cell Culture and Therapy Unit, Hôpital La Conception, Hôpitaux Universitaires de Marseille) were grown in 0.1% gelatin-coated flasks with serum-free endothelial growth medium-2 (EGM2, Lonza, Bale, Switzerland) in a humidity-saturated incubator, under 5% CO_2_ at 37 °C. Conditioned medium of HUVECs was collected after 24 h of stimulation with tumor necrosis factor alpha (TNF-alpha, 10 ng·mL^−1^_,_ Merck Sigma-Aldrich, St Quentin Fallavier, France) to increase vesiculation. After two initial centrifugations to discard debris (300× *g*, 5 min) and apoptotic bodies (2000× *g*, 15 min), clarified conditioned medium was subjected to differential ultracentrifugation (70,000× *g*; 90 min at 4 °C, Aventi J30-I, JA-30.50T1 rotor, Beckman-Coulter, Villepinte, France) to obtain LEVs. The resultant LEV pellet was washed twice in 30 mL of Ca^2+^-/Mg^2+^-free phosphate buffer saline solution (PBS Gibco, ThermoFisher, Waltham, MA, USA) in the same conditions.

### 2.2. Purification and Characterization of LEVs

#### 2.2.1. Purification

A single-use SEC column (qEV single, Izon, Oxford, UK) was preconditioned with “binding buffer” solution (0.1 mol·L^−1^ HEPES/NaOH, pH 7.4, 140 mmol·L^−1^ NaCl, 25 mmol·L^−1^ CaCl_2_, 0.22 µm filtered). LEVs, obtained as described above, were then loaded onto the column, and eluted according to the manufacturer’s recommendations, recovering successive fractions as follows: V_0_ 1000 μL (dead volume); V_1_ 600 μL (expected LEV-containing fraction); V_2_ 600 μL; V_3_ 600 μL; V_4_ 1000 μL; V_5_ 1000 μL; V_6_ 1000 μL (V_2_–V_6_: washing). Further quantitative (counting) and qualitative characterizations (size distribution, morphology, protein content, and phenotyping) were performed on V_1_-eluted LEVs after SEC purification.

#### 2.2.2. Flow Cytometry

LEV samples were analyzed by high-sensitivity flow cytometry using a standardized Gallios flow cytometer (Beckman Coulter, Villepinte, France) as previously described [28]. Briefly, a 1:500-diluted 30 µL sample was incubated for 15 min with an appropriate amount of specific antibody and 10 µL of Annexin V-FITC (AnnV-FITC, Tau Technologies, Kattendijke, The Netherlands). Binding buffer solution (400 µL, as described in Section 2.2.1) was then added to improve the binding of AnnV to phosphatidylserines. LEV count beads (30 µL, Biocytex, Marseille, France) were added to determine the concentration of LEVs in each sample. The flow cytometer settings and LEV gating were performed with Megamix beads (Biocytex, Marseille, France). Positive-AnnV-LEVs were defined as total of LEVs. Specific fluorescent antibodies (ICAM-1-directed CD54-PE, #IM1239V; CD31-PE, #PN-IM2409 and CD146-PE, #AD7483; Beckman Coulter, Villepinte, France) were used to characterize LEV surface antigens. Analysis was performed with Kaluza Analysis software 1.2 (Beckman Coulter, Villepinte, France), as previously described [29]. A total 10^8^ LEV samples were stored at −80 °C until further use.

#### 2.2.3. Tunable Resistive Pulse Sensing (TRPS)

TRPS was performed using a qNano Gold TRPS measurement instrument (Izon, Oxford, UK) and CPC400 calibration beads with a mean diameter of 350 nm as calibration standard, following the manufacturer’s instructions [30]. Samples were diluted in PBS or HEPES buffer solutions in a small sterile tube and analyzed using a 200–1000 nm NP400 nanopore (Izon, Oxford, UK) at a stretch of 43–45 mm. Voltage was set on 0.30–0.50 V to achieve a stable 110–130 nA current and a 1.4–2.0 kPa pressure, with root mean square noise below 10 pA. Blockade counts setting in this study was fixed at minimum of 500 vesicles count for each, and each sample was analyzed in duplicate. Data were collected and analyzed using Izon Control Suite software v3.3.3.2001 (Izon, Oxford, UK).

#### 2.2.4. Transmission Electron Microscopy (TEM)

LEV pellets were fixed in 2% paraformaldehyde, 2.5% glutaraldehyde overnight, post-fixed in 2% osmium for 1 h on ice, dehydrated in gradient series of acetone baths, and embedded in epoxy resin. Pellets were sectioned on an UC7 ultra-microtome (Leica, Wetzlar, Germany), and sections were contrasted with aqueous uranyl acetate 1% (10 min) and lead citrate (4 min). The grids were observed at 80 kV on a FEI Morgagni transmission electron microscope (ThermoFisher, Waltham, USA) and images were acquired using a MegaView3 camera (Emsis, Muenster, Germany).

#### 2.2.5. Western Blot Analysis

LEV pellets were suspended in radioimmunoprecipitation assay buffer solution (20 µL, Pierce ThermoFisher, Waltham, MA, USA) on ice for 15 min. Samples were centrifuged at 14,000× *g* for 15 min at 4 °C. Proteins were separated on 4–12% gradient sodium-dodecylsulfate/polyacrylamide gel, and blotted on nitrocellulose C+ membranes (Amersham Protran, Merck Sigma-Aldrich, St Quentin Fallavier, France). Equal loading was verified using Ponceau Red solution (P7170, Merck Sigma-Aldrich). Membranes were blocked in 3% bovine serum albumin/tris-buffered saline (TBS, ET220B, Euromedex, Souffelweyersheim, France), for 1 h at room temperature (RT), before proceeding with the antibody incubation. All primary antibody incubations were performed in blocking buffer overnight at 4 °C at the following dilutions: CD51 (1/1000, #PA527272), β_3_-integrin (1/1000, #336402), albumin (1/1000, #MA5-32531), CD63 (1/1500, #10628D), β-tubulin (1/1000, #2128S), β-actin (1/1000, #8457L), CD81 (1/800, #10630D), and caveolin-1 (1/1000, #03-600). Antibodies against CD51, albumin, CD63, CD81, and caveolin-1 were purchased from ThermoFisher Scientific (Waltham, MA, USA). The antibody against β_3_-integrin antibody was purchased from Biolegend (San Diego, CA, USA). Membranes were washed three times with TBS/Tween 0.1% (P1379, Merck Sigma-Aldrich) for 10 min, followed by an incubation with secondary antibodies. Horseradish peroxidase-conjugated, anti-mouse or anti-rabbit antibodies (#31430, #31460, Thermo-Fisher, Waltham, MA, USA) were used as secondary antibodies (1/2000) and incubated for 1 h at room temperature. Immunocomplexes were visualized by chemiluminescence using ECL Pierce substrate (Thermofisher, Waltham, MA, USA), following the manufacturer’s instructions. The G-BOX Imaging System (GeneSys, Syngene, Cambridge, UK) was used to delimit specific bands. After initial immunodetection, membranes were stripped from antibodies and re-probed with antibody against total protein or another protein with the same molecular weight. All proteins for each panel were assessed on one membrane; therefore, actin expression needed to be determined only once to control loading.

### 2.3. Radiolabeling of LEVs

Radiolabeling of LEVs was achieved using [^99m^Tc]Tc-Annexin-V-128 (AnnV), a phosphatidylserine-targeting radiotracer for single photon emission-computed tomography (SPECT). The radiotracer was prepared by radiolabeling Annexin-V-128 lyophilizate (Advanced Accelerator Applications, Saint Genis Pouilly, France) with a fresh [^99m^Tc]TcO_4_^-^ pertechnetate solution (740 MBq/100 μL) eluted from a commercial [^99^Mo]Mo/[^99m^Tc]Tc generator (Tekcis, Curium, Paris, France). After gentle stirring, the solution was incubated for 90 min at room temperature. Radiochemical purity was assessed by instant thin layer radiochromatography using a radiochromatograph (miniGITA, Elysia-Raytest, Straubenhardt, Germany), Whatman paper (Cytiva, Marlborough, MA, USA) as stationary phase, and citric acid–citrate–dextrose solution (ACD/A solution, Fresenius-Kabi, Paris, France) as mobile phase. A radiochemical purity above 95% enabled to validate the radiolabeling. A measure of 200 MBq/100 μL of radiotracer was mixed with 15 μL of concentrated binding buffer solution (140 mmol·L^−1^ NaCl; 25 mmol·L^−1^ CaCl_2_; 10 mmol·L^−1^ HEPES and pH 7.4), added to a sample of 100.10^6^/50 μL LEVs, leading to a final volume of 165 µL and incubated for 20 min at room temperature. 

### 2.4. Purification of Radiolabeled LEVs from Free [^99m^Tc]Tc-AnnV Radiotracer

To further purify radiolabeled [^99m^Tc]Tc-AnnV-LEVs from free [^99m^Tc]Tc-AnnV radiotracer, the elution profile from qEV single-use SEC of radiolabeled [^99m^Tc]Tc-AnnV-LEVs was first compared to that of non-radiolabeled LEVs. Three samples containing [^99m^Tc]Tc-AnnV-LEVs and 3 samples containing non-radiolabeled LEVs were loaded on qEV single-use SECs and eluted in the same operating conditions as for LEV purification described supra. LEVs were quantified in each eluted fraction by flow cytometry as described supra.

Then, 3 samples containing [^99m^Tc]Tc-AnnV-LEVs and 3 samples containing free [^99m^Tc]Tc-AnnV only were loaded on qEV single-use SECs and eluted in the same operating conditions as for LEV purification described supra. The activity in V_1_ was measured in a dose calibrator (Scintidose, LemerPax, La Chapelle sur Erdre, France).

### 2.5. Stability of Radiolabeled LEVs in Serum

A 40 µL sample of purified radiolabeled LEVs was mixed with 60 µL of human serum and incubated for 0 or 30 min at 37 °C (*n* = 6 each). The total 100 µL were then re-purified by SEC. The activity in V_1_ fraction was measured in a dose calibrator (Scintidose, LemerPax, La Chapelle sur Erdre, France).

### 2.6. In Vivo Experimentations

**Figure 1:**
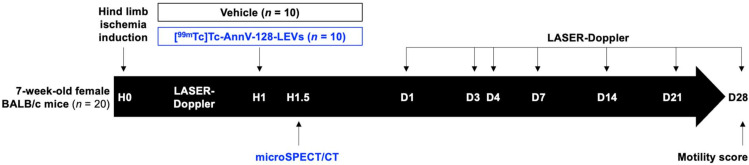
Experimental paradigm. Twenty mice underwent hind limb ischemia induction surgery. the hind limb perfusion was quantified by LASER Doppler allowing the constitution of 2 homogeneous groups of 10 mice receiving either [^99m^Tc]Tc-AnnV-LEVs or the vehicle (calcic binding buffer as described in Section 2.2.1). A subgroup (*n* = 3) from the Vehicle group received free [^99m^Tc]Tc-AnnV. MicroSPECT/CT was performed 30 min after the injection of radiolabeled compounds. All the mice were followed up by LASER Doppler for their hind limb perfusion on days 1, 3, 4, 7, 14, 21, and 28. A motility impairment score was calculated on day 28.

Procedures using animals were approved by the Institution’s Animal Care and Use Committee (Project #14177, CE71 Aix-Marseille University) and were conducted according to the 2010/63/EU European Union Directive and following the ARRIVE 2.0 guidelines [31], by qualified and trained operators in an accredited laboratory (A-13-055-32). A total of 20 female 7-week-old BALB/c mice (Janvier Labs, France) were housed in enriched cages placed in a temperature- and hygrometry-controlled room with a daily monitoring, fed with water and commercial diet ad libitum, and weighed once a week. No animal was excluded during the 28-day follow-up. In vivo experimentations are summarized in experimental paradigm (Figure 1).

### 2.7. Mouse Model of Hind Limb Ischemia Induction and Follow-Up

Unilateral hind limb ischemia was performed after femoral artery excision as previously described [32]. LASER Doppler perfusion imaging (Perimed, Craponne, France) was performed to quantify hind limb perfusion on a 37 °C heated bed under isoflurane anesthesia (induction at 5%, maintenance at 1.5% in air, Iso-vet, Piramal). Each LASER Doppler acquisition lasted 120 s and was repeated 3 times for each animal. On day 0, LASER Doppler was used to check and quantify the induced perfusion defect in the right hind limb, allowing the constitution of 2 homogeneous groups of 10 mice accordingly, for subsequent experiments. Hind limb perfusion was then quantified on days 1, 3, 7, 14, 21, and 28 post-ischemia. Results were expressed as a mean ± SD ratio of ischemic-to-contralateral (i/c) hind limb blood flow, and graphically represented as a mean ± SD reperfusion ratio to day 0. A motility impairment score, inspired by Suffee et al., and as previously published [32,33], was calculated for each mouse on day 28, as follows: 1—unrestricted active movement; 2—restricted active foot; 3—use of the other leg only; 4—leg necrosis; 5—self-amputation.

### 2.8. Quantification of the In Vivo Biodistribution of Radiolabeled LEVs by Isotopic Imaging

Micro-single photon emission computed tomography coupled with micro-tomodensitometry (microSPECT/CT) imaging sessions were performed on a NanoSPECT/CT+ camera (Mediso, Budapest, Hungary). One group received an injection of radiolabeled LEVs ([^99m^Tc]Tc-AnnV-LEVs, 2.0 ± 0.5 × 10^6^ LEVs/2.0 ± 0.4 MBq/150 µL, *n* = 10) in the caudal vein. The other group was injected in the caudal vein with a solution containing only the vehicle (150 µL binding buffer as described in Section 2.2.1, *n* = 10). A subgroup of the control group received an injection in the caudal vein of 7.1 ± 0.7 MBq/50 µL [^99m^Tc]Tc-AnnV (*n* = 3). All the 20 mice, whether injected with radioactive materials or not, were anesthetized under isoflurane (induction at 5%, maintenance at 1.5%) on a heated bed, to be equally exposed to anesthesia. Subsequent experiments and data analysis were performed by blind operators. Only the 3 mice injected with free [^99m^Tc]Tc-AnnV and the 10 mice injected with [^99m^Tc]Tc-AnnV-LEVs underwent a 15 min whole-body microSPECT/CT acquisition starting 30 min after the injection. The animals were constantly monitored for breathing during the acquisition. Quantitative region of interest analysis of the SPECT signal was performed using Invivoscope software (Invicro, Boston, MA, USA) to quantify the tissue uptake of [^99m^Tc]Tc-AnnV-LEVs and that of free [^99m^Tc]Tc-AnnV. Tissue uptake values were expressed as a mean ± SD percentage of injected dose per cubic millimeter of tissue (%ID/mm^3^) as recommended by the AQARA requirements [34]. The SPECT signal quantifications expressed as percentage of injected dose can be found in Supplementary Figure S1.

### 2.9. Statistical Analysis

Data were analyzed using Prism v9.1 software (GraphPad, San Diego, CA, USA). The collected activities from SEC in V_1_ and the radiolabeling stability over time in serum were analyzed using an unpaired t-test. The differences between quantified activities in each organ or hind limb from each condition were compared using a two-way ANOVA followed by Šídák’s multiple comparison post hoc test. Animal weight and LASER Doppler over time were analyzed with a two-way repeated-measures ANOVA followed by a Šídák’s multiple comparisons post hoc test. Correlation between LEV homing and late LASER Doppler data was tested using Pearson *R* test after validating data for normality. Sample size was calculated using the BiostaTGV online tool (https://biostatgv.sentiweb.fr, accessed on 23 March 2018). Samples distributions were tested for normality using the Shapiro–Wilk test. Unless indicated otherwise, data were expressed as mean ± SD values, *p* < 0.05 indicating statistical significance.

## 3. Results

### 3.1. Characterization of Produced Endothelial LEVs

**Figure 2:**
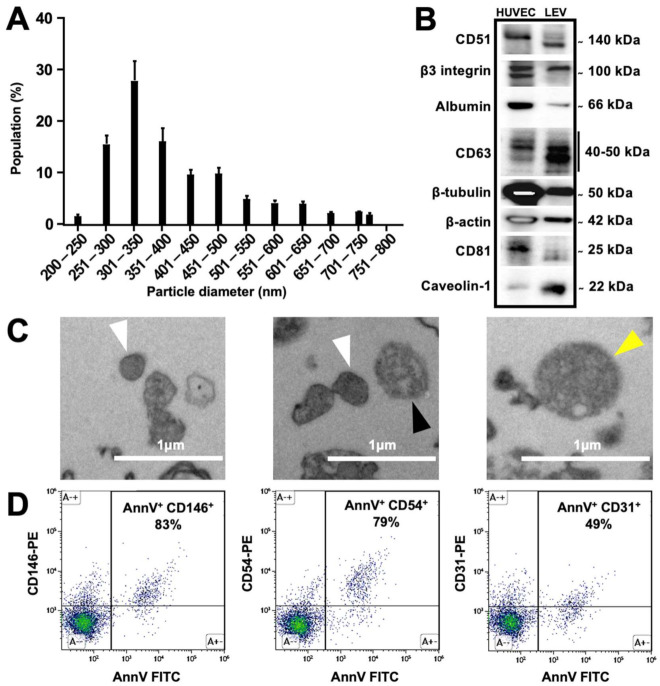
(**A**) Description of LEV size by tunable resistive pulse sensing (TRPS). TRPS size distribution histogram of LEVs released by HUVEC cells exposed for 24 h to TNF. Bars represent the mean ± SD (*n* = 2). (**B**) Characterization of LEVs using Western blotting for the presence of LEV protein markers (~1.5 × 10^8^ LEVs per lane). Blot images are presented from different parts of the same membrane. (**C**) Representative transmission electronic microscopy image of LEVs isolated after SEC. White and black arrowheads pointed EV sized 200–300 nm and 300–500 nm, respectively. Yellow arrowhead denotes EVs sized 500–800 nm (*n* = 2). (**D**) Flow cytometric characterization of LEVs after calibration with fluorescent silica beads. EVs were defined as phosphatidylserine-exposing events in the LEV gate (CD146^±^, CD31^±^, and ICAM1/CD54^±^ population).

Biophysical characterization by TRPS showed approximately 60% of the LEVs ranged between 250 and 400 nm (Figure 2A). Western blot analysis identified protein markers of LEVs (Figure 2B). CD63 and CD81 tetraspanins, predominantly associated with late endocytic organelles and the smallest LEV types [13,35], were present, respectively, as 40–50 kDa and 25 kDa bands. CD51 integrin (alpha-V) was present, whereas β_3_-integrin was abundant, as previously described [36]. Caveolin-1, identified as a major component of LEVs and involved in diverse protein trafficking pathways [13,37,38], was abundantly present, as were actin and tubulin. Albumin, a major constituent of non-LEV structures [13], appeared as a weak band, indicating the low level of major cellular contaminants on the isolated LEV preparations. TEM analysis showed circular LEVs with typical bilayer membrane with a mean size of 434.5 ± 124.20 nm (*n* = 2) (Figure 2C). Isolated, SEC-purified LEV fractions from a single donor were characterized by flow cytometry regarding the exposure of phosphatidylserine and markers of cellular origin. The expression of these markers was also evaluated, and LEVs were stained with a combination of antibodies directed against endothelial markers (CD31 and CD146), as well as the activation marker ICAM1/CD54. Approximately half of the HUVEC-derived LEVs stained for CD31, while most stained strongly for CD146 and for ICAM1/CD54 (Figure 2D). Altogether, these data confirmed the endothelial cell origin of LEVs.

### 3.2. Endothelial LEVs Were Successfully Radiolabeled and Purified

**Figure 3:**
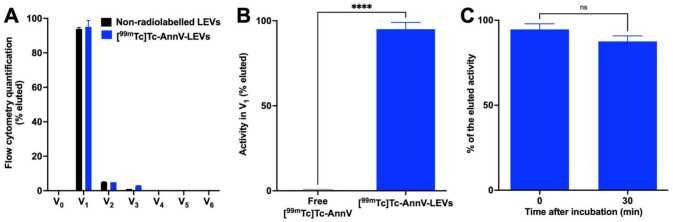
Radiolabeling, purification and stability of radiolabeled LEVs. (**A**) Flow cytometry quantifications of non-radiolabeled LEVs (black bars) and radiolabeled [^99m^Tc]Tc-AnnV-LEVs (blue bars) in elution fractions (V_#_) from qEV SEC column. (**B**) Dose calibrator measurement of the activity of free [^99m^Tc]Tc-AnnV radiotracer or [^99m^Tc]Tc-AnnV-LEVs in V_1_ elution fraction from qEV SEC column (**** *p* < 0.0001, *n* = 3). (**C**) Radiolabeling stability of [^99m^Tc]Tc-AnnV-LEVs in serum up to 30 min after incubation (*p* = 0.0560, *n* = 3).

[^99m^Tc]Tc-AnnV was prepared with a 97 ± 2% radiochemical purity. Size-exclusion chromatography (SEC) column enabled the purification of radiolabeled [^99m^Tc]Tc-AnnV-LEVs from free [^99m^Tc]Tc-AnnV radiotracer in V_1_ elution fraction (95.1 ± 4.0% and 0.4 ± 0.5%, respectively, **** *p* < 0.0001, *n* = 3, Figure 3A,B). The radiolabeling stability was validated up to 30 min after incubation in vitro (0 min: 94.7 ± 3.3%; 30 min: 87.6 ± 3.4%; *p* = 0.0560, *n* = 3, Figure 3C).

### 3.3. Endothelial LEVs Preferentially Homed to the Ischemic Hind Limb

**Figure 4:**
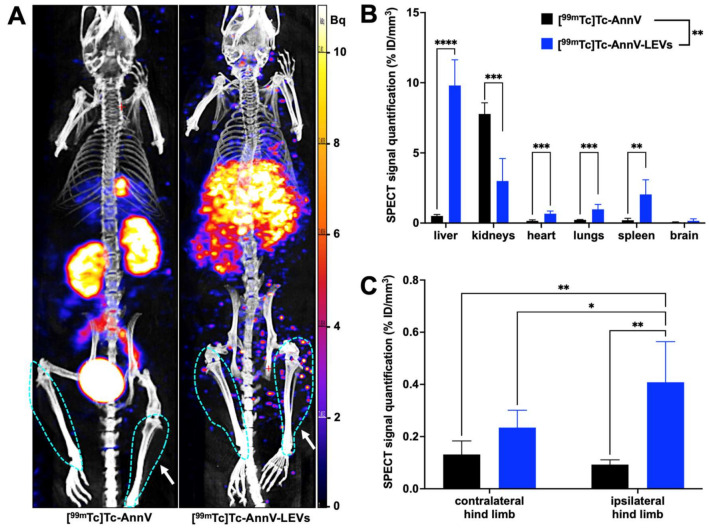
Quantification of radiolabeled LEV biodistribution by microSPECT/CT imaging in a mouse model of hind limb ischemia. (**A**) Representative maximum intensity projection images of free [^99m^Tc]Tc-AnnV biodistribution (left) and radiolabeled [^99m^Tc]Tc-AnnV-LEV biodistribution (right) by microSPECT/CT imaging 30 min after injection in a mouse model of hind limb ischemia (the blue dots delimiting regions of interest, ipsilateral hind limb on the right side of each animal, pointed by the arrow). (**B**,**C**) MicroSPECT/CT signal quantifications of free [^99m^Tc]Tc-AnnV biodistribution (black bars, *n* = 3) and [^99m^Tc]Tc-AnnV-LEV biodistribution (blue bars, *n* = 10) 30 min after injection (* *p* < 0.05; ** *p* < 0.01; *** *p* < 0.001; **** *p* < 0.0001).

MicroSPECT/CT biodistributions of free [^99m^Tc]Tc-AnnV and [^99m^Tc]Tc-AnnV-LEVs 30 min after injection were overall highly significantly different (two-way ANOVA ** *p* = 0.0019, Figure 4A,B), especially in the liver, in the kidneys, in the heart, in the lungs, and in the spleen (Table 1, Figure 4B). A significantly higher SPECT signal quantification was found with [^99m^Tc]Tc-AnnV-LEVs in the ischemic hind limb compared with the contralateral hind limb 30 min after injection (* *p* = 0.0090); whereas, no significant difference was found between SPECT signal quantifications in the ischemic and in the contralateral hind limbs with free [^99m^Tc]Tc-AnnV (*p* = 0.9722). A significantly higher SPECT signal quantification was found in the ischemic hind limb with [^99m^Tc]Tc-AnnV-LEVs compared with that of free [^99m^Tc]Tc-AnnV (** *p* = 0.0013, Table 1, Figure 4C).

### 3.4. Tracking of LEV Homing Correlated with Therapeutic Effects

**Figure 5:**
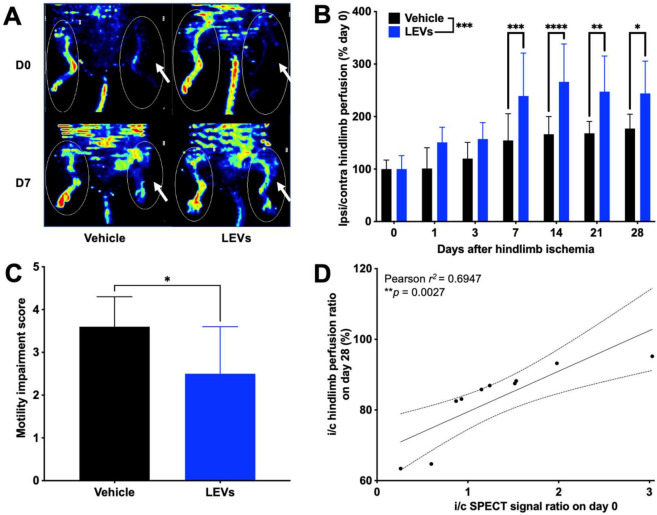
(**A**) Representative LASER Doppler perfusion imaging showing the recovery of blood perfusion in the ischemic hind limb (white arrow: ischemic hind limb). (**B**) Quantitative analysis expressed as ischemic-to-contralateral muscle ratio normalized to day 0 (%, mean ± SD) from day 0 to day 28 in vehicle- (*n* = 10, black bars) or LEV-treated mice (*n* = 10, blue bars) (* *p* < 0.05; ** *p* < 0.01; *** *p* < 0.001; **** *p* < 0.0001). (**C**) Motility impairment score on day 28 in vehicle- (*n* = 10, black bars) or LEV-treated mice (*n* = 10, blue bars): 1—unrestricted active movement; 2—restricted active foot; 3—use of the other leg only; 4—leg necrosis; 5—self-amputation (* *p* < 0.05). (**D**) Positive correlation between the ipsilateral-to-contralateral [^99m^Tc]Tc-AnnV-LEVs SPECT signal on the day of ischemia and the ipsilateral-to-contralateral hind limb perfusion on day 28. Pearson *r^2^* = 0.4108, * *p* = 0.0458.

An earlier and higher angiogenic activation was found in LEV-treated mice, using [^68^Ga]Ga-RGD_2_ microPET/CT (Supplementary Figure S2). Blood flow recovery was overall significantly different from one condition to another (two-way RM ANOVA *** *p* = 0.0003, *n* = 10 per condition): a significantly higher i/c LASER Doppler signal ratio normalized to day 0 was observed in the LEV-treated group compared with vehicle-treated group on day 7 (respectively, 239.3 ± 81.6% and 154.5 ± 50.9%, *n* = 10, *** p = 0.0006), on day 14 (respectively, 266.2 ± 72.1% and 166.4 ± 33.7%, *n* = 10, **** *p* < 0.0001), on day 21 (respectively, 247.6 ± 67.8% and 168.1 ± 22.6%, *n* = 10, ** *p* = 0.0015), and on day 28 after ischemia (respectively, 244.1 ± 61.5% and 177.2 ± 27.3%, *n* = 10, * *p* = 0.0119) (Figure 5A,B). Quantitative analysis of LASER Doppler signal expressed as ischemic-to-contralateral muscle ratio (%, mean ± sd) from day 0 to day 28 are presented in Supplementary Figure S3. A significantly lower motility impairment score was observed in the LEV-treated group on day 28 (2.5 ± 1.1) compared with that of the vehicle-treated group (3.6 ± 0.7; * *p* = 0.0157, *n* = 10) (Figure 5C). A significant, positive correlation was found between the i/c [^99m^Tc]Tc-AnnV-LEVs SPECT signal on the day of their injection, and the late i/c hind limb perfusion assessed by LASER Doppler on day 28 (Pearson *r^2^* = 0.6947, ** *p* = 0.0027) (Figure 5D).

## 4. Discussion

A simple method was designed for radiolabeling endothelial LEVs using [^99m^Tc]Tc-AnnV. In a preclinical model of hind limb ischemia, early and specific homing of radiolabeled LEVs to the ischemic hind limb were quantified on the day of ischemia and positively correlated with reperfusion intensity at a later stage on day 28 after ischemia, associated with an improved motility function.

SEVs are described as being very quickly cleared from the plasma, within the first 30 min following their injection [37,39]. By extrapolation to LEVs and to avoid the potential risk of quantifying an unspecific signal related to LEV degradation and free [^99m^Tc]Tc-AnnV-128 tissue accumulation at later time points, the radiolabeling stability was validated up to 30 min after incubation in vitro and the quantification of microSPECT/CT signal in main organs and hind limbs was performed up to 30 min after injection. In line with previous reports on the biodistribution of extracellular vesicles with other techniques, [^99m^Tc]Tc-AnnV-LEVs were mainly distributed in organs of the mononuclear phagocyte system with highest accumulation in the liver, secondarily in kidneys, spleen, and lungs.

Remarkably, the [^99m^Tc]Tc-AnnV-LEVs SPECT signal quantified in the ischemic hind limb was 4.5 times higher than that of free [^99m^Tc]Tc-AnnV in the same hind limb, and 2 times higher than that of [^99m^Tc]Tc-AnnV-LEVs in the contralateral hind limb. Of note, apoptosis, that can be imaged with [^99m^Tc]Tc-AnnV radiotracer, occurs in later days in the chronology of ischemic hind limb model and is very unlikely to generate a [^99m^Tc]Tc-AnnV uptake on the day of ischemia [38,40]. Most importantly, despite an unfavorable input function in the ischemic hind limb on the day of ischemia, a quick and significant homing of LEVs was quantified in the ischemic region. As for many cell-based therapies, the very small proportion of injected radiolabeled LEVs reaching the ischemic site (<1% of the injected dose) was nevertheless sufficient to induce significant therapeutic effects [41,42]. The administration route directly influences the biodistribution of extracellular vesicles and constitutes an interesting avenue for refinement; Wiklander et al. reported subcutaneous and intraperitoneal delivery routes associated with half less accumulation of the extracellular vesicles in the liver compared with intravenous delivery route [39]. Indeed, diffusion and transport of extracellular vesicles is influenced by their environment and the specific shear stress in the impaired locoregional blood circulation passively affects their uptake [43,44].

Most outstandingly, no later than 7 days after the insult, LEVs enabled a significant earlier and higher vascular recovery. Besides, the more LEVs were addressed to the site of ischemia on the day of their injection, the better the vascular recovery 28 days after was, corroborating the importance of promoting early angiogenesis in post-ischemic regenerative therapies [45]. Preclinical ex vivo evidences of post-ischemic recovery enhancement following LEV injection are in line with observations in the literature [46,47,48]. The therapeutic properties of endothelial LEVs rely not only on their origin, but also on the considered animal disease model or the patient’s condition [16,49]: for instance, extracellular vesicles from human adipose tissue were also recently reported with pro-angiogenic properties and could have a high potential for therapeutic use in ischemia [50]. Considering the inner composition and phenotype of LEVs, but also considering the route of administration, each LEV subtype is expected to demonstrate a different biodistribution profile (liver retention and non-specific accumulation in healthy tissues) linked to various homing and various therapeutic effects (uptake in target tissues) [39]. Consequently, such a biodistribution study should be repeated for each LEV subtype, testing for a positive correlation between their early homing and the later therapeutic effects, for each disease model or pathological condition. Of note, the therapeutic performance of LEVs related in this study might be underestimated due to the use of human cell-derived LEVs instead of mice cell-derived LEVs, mainly because the primary objective was to set up and demonstrate the feasibility of a radiolabeling method for evaluating the biodistribution of human LEVs in clinical trials. The absence of a LEV co-labeling method and of a supplementary method to radionuclide tracking could be considered as a limitation of our study, although hardly technically feasible in vivo regarding the short life of LEVs. Notably, as this LEV radiolabeling method with [^99m^Tc]Tc-AnnV relies on an industrial GMP-manufactured radiotracer, this radiolabeling method could be easily transferable to other LEV subtypes and most importantly, would be an asset as companion tool for emerging therapies based on extracellular vesicles at the clinical stage.

## 5. Conclusions

This work reported an innovative method to radiolabel endothelial LEVs enabling quantification of their in vivo biodistribution after systemic injection. Applied to a mouse model of critical hind limb ischemia, microSPECT/CT imaging enabled the quantification of an early and specific homing of LEVs to ischemic tissues that correlated with reperfusion intensity 28 days after ischemia. LEV injection was also associated with an enhanced motricity on day 28. This concept could be a major asset for investigating the biodistribution of LEVs issued from other cell types, including cancer, thus partly contributing to better knowledge and understanding of their fate after injection.

## Figures and Tables

**Table 1:** Quantification of microSPECT/CT signal in main organs and hind limbs, and comparison of SPECT signal quantifications between free [^99m^Tc]Tc-AnnV and [^99m^Tc]Tc-AnnV-LEVs (*p* value line).

%ID/mm^3^	Liver	Kidneys	Heart	Lungs	Spleen	Brain	Ipsi Hind Limb	Contra Hind Limb
**free [^99m^Tc]Tc-AnnV** **(*n* = 3)**	0.51 ± 0.10	7.78 ± 0.79	0.16 ± 0.07	0.23 ± 0.02	0.20 ± 0.14	0.05 ± 0.04	0.09 ± 0.02 ^€^	0.13 ± 0.05 ^€^
**[^99m^Tc]Tc-AnnV-LEVs** **(*n* = 10)**	9.81 ± 1.83	2.99 ± 1.60	0.67 ± 0.19	0.97 ± 0.36	2.04 ± 1.05	0.16 ± 0.14	0.41 ± 0.09 ^¥^	0.23 ± 0.06 ^¥^
**Post**-**hoctest *p* value**	**** <0.0001	*** 0.0010	*** 0.0003	*** 0.0005	** 0.0019	ns 0.2957	** 0.0013	ns 0.4958

^€^ Comparison of free [^99m^Tc]Tc-AnnV SPECT signal quantifications in ipsilateral to contralateral hind limbs: *p* = 0.9722, ns; ^¥^ comparison of [^99m^Tc]Tc-AnnV-LEVs SPECT signal quantifications in ipsilateral to contralateral hind limbs: *p* = 0.0090 (**) (** *p* < 0.01; *** *p* < 0.001; **** *p* < 0.0001).

## Data Availability

The data presented in this study are available on request from the corresponding author.

## Supplementary Materials

The following supporting information can be downloaded at: https://www.mdpi.com/article/10.3390/pharmaceutics14010121/s1. Figure S1: SPECT signal quantification in organs and hind limbs expressed as percentage of the injected dose (%ID). Figure S2: Angiogenic activation in a mouse model of hindlimb ischemia treated by LEVs or vehicle. Figure S3: Quantitative analysis of LASER Doppler signal expressed as ischemic-to-contralateral muscle ratio (%, mean ± sd) from day 0 to day 28.
