# Supplementary material for: Tracking Radiolabeled Endothelial Microvesicles Predicts Their Therapeutic Efficacy: A Proof-of-Concept Study in Peripheral Ischemia Mouse Model Using SPECT/CT Imaging
Source: Pharmaceutics. 2022 Jan 4;14(1):121. doi: 10.3390/pharmaceutics14010121 (PMC8778059; doi:10.3390/pharmaceutics14010121)
Supplement: Supplementary file 1 [file pharmaceutics-14-00121-s001.zip › pharmaceutics-1513903-supplementary.pdf]

pharmaceutics

MDPI

Supplementary Materials: Tracking Radiolabeled Endothelial Microvesicles Predicts Their Therapeutic Efficacy: A Proof-of-Concept Study in Peripheral Ischemia Mouse Model Using SPECT/CT Imaging

Romain Giraud, Anaïs Moyon, Stéphanie Simoncini, Anne-Claire Duchez, Vincent Nail, Corinne Chareyre, Ahlem Bouhlel, Laure Balasse, Samantha Fernandez, Loris Vallier, Guillaume Hache, Florence Sabatier, Françoise Dignat-George, Romaric Lacroix, Benjamin Guillet and Philippe Garrigue

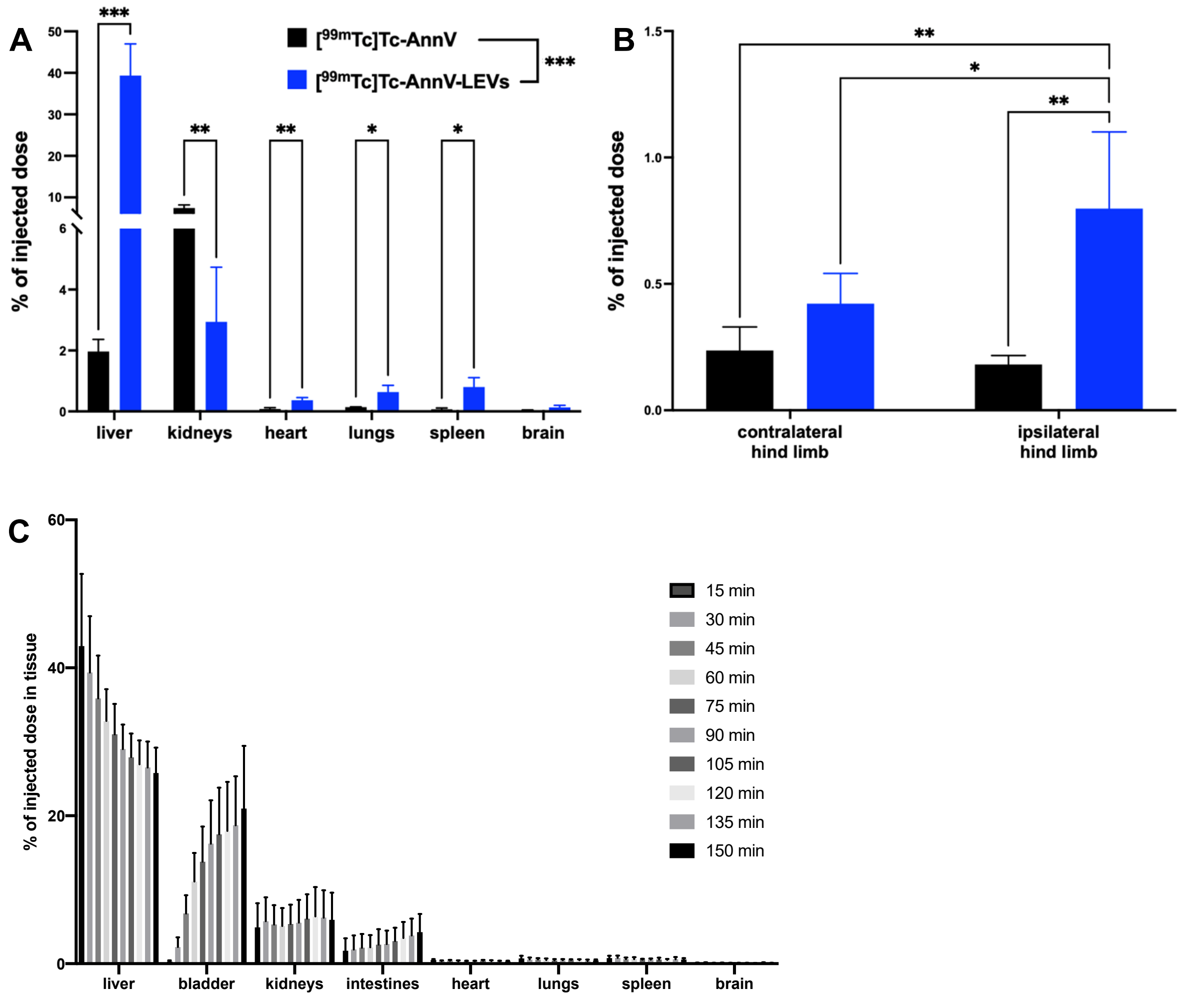

Figure S1. SPECT signal quantification in organs and hind limbs expressed as percentage of the injected dose (%ID). A and B: MicroSPECT/CT signal quantifications of free [^{99m}Tc]Tc-AnnV biodistribution (black bars, n = 3) and [^{99m}Tc]Tc-AnnV-LEV biodistribution (blue bars, n=10) 30 min after injection (*P < 0.05; **P < 0.01; ***P < 0.001; ****P < 0.0001). **C:** MicroSPECT/CT signal quantifications of [^{99m}Tc]Tc-AnnV-LEV biodistribution (n = 10) each 15 min, from 15 min post-injection to 150 min post-injection. The rapidly increasing signal quantified in the bladder from 45 min after injection presumes in vivo degradation of LEVs.

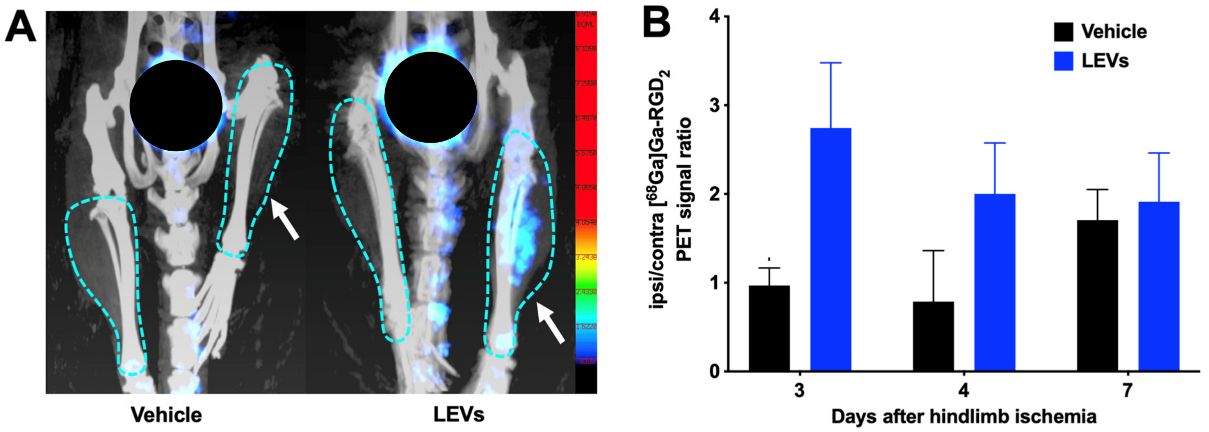

Figure S2. Angiogenic activation in a mouse model of hindlimb ischemia treated by LEVs or vehicle. **(A)** Representative images of [^{68}Ga]Ga-RGD$_2$ microPET/CT on day 3 after ischemia in vehicle- (n = 3, black bars) or LEV-treated mice (n = 3, blue bars). The blue dots delimit regions of interest, ipsilateral hind limb on the right and contralateral hind limb on the left side of each animal. **(B)** [^{68}Ga]Ga-RGD$_2$ microPET/CT quantifications on days 3, 4 and 7 after ischemia.

Follow-up and quantification of angiogenic activation by [^{68}Ga]Ga-RGD$_2$ microPET/CT imaging

[^{68}Ga]Ga-RGD$_2$ was prepared by mixing 20 µg of NODAGA-RGD dimer peptide (RGD$_2$, ABX Biochemicals) with 150 µL of 4.0 mol·L^{-1} ammonium acetate buffer, then with 500 µL of a fresh [^{68}Ga]GaCl$_3$ solution eluted with pharmaceutical-grade 0.1 mol·L^{-1} hydrochloric acid (Eckert&Ziegler, Berlin, Germany) from a pharmaceutical-grade [^{68}Ge]Ge/[^{68}Ga]Ga generator (Galliapharm®, Eckert&Ziegler, Berlin, Germany), at room temperature for 5 min. Radiochemical purity was assessed by instant thin layer radiochromatography using a radiochromatograph (miniGITA, Elysia-Raytest, Straubenhardt, Germany), iTLC-SG paper (Agilent, Santa Clara, USA) as stationary phase, a freshly prepared 0.1 mol.L^{-1} sodium citrate pH 5 solution as mobile phase A (Rf_0: [^{68}Ga]Ga-RGD$_2$ + [^{68}Ga]Ga-colloids; Rf_1: free [^{68}Ga]GaCl$_3$) and a 50:50 mixture of freshly prepared 1 mol·L^{-1} ammonium acetate solution with methanol as mobile phase B (Rf_0: free [^{68}Ga]GaCl$_3$ + [^{68}Ga]Ga-colloids; Rf_1: [^{68}Ga]Ga-RGD$_2$). A radiochemical purity above 95% enabled to validate the radiolabeling. Mice (n = 3 per condition) were injected with 6.0 ± 1.1 MBq/50 µL of [^{68}Ga]Ga-RGD$_2$ in the caudal vein on days 3, 4, and 7 post-ischemia. Twenty-minute-long micro-Positron Emitting Tomography coupled with Computed Tomography (microPET/CT) images were acquired 60 min after [^{68}Ga]Ga-RGD$_2$ injection on a NanoscanPET/CT camera (Mediso, Budapest, Hungary) under 1.5% isoflurane anesthesia. Quantitative ROI analysis of PET signal was performed on attenuation- and decay-corrected PET images using InterviewFusion software (Mediso, Budapest, Hungary) and tissue uptake values were expressed as a mean±sd ischemic-to-contralateral (i/c) hindlimb signal ratio.

Endothelial LEV administration was associated with earlier angiogenic activation.

As soon as day 3 post-ischemia, [^{68}Ga]Ga-RGD$_2$ microPET/CT quantifications showed a higher i/c radiotracer uptake ratio in LEVs-treated animals compared to vehicle group (respectively 2.7 ± 0.7 and 0.9 ± 0.2; n = 3) maintained up on day 4 (respectively 2.0 ± 0.6 and 0.8 ± 0.6, n = 3). Both groups presented an elevated i/c radiotracer uptake ratio on day 7 (vehicle: 1.7 ± 0.3, LEVs: 1.9 ± 0.6, n = 3).

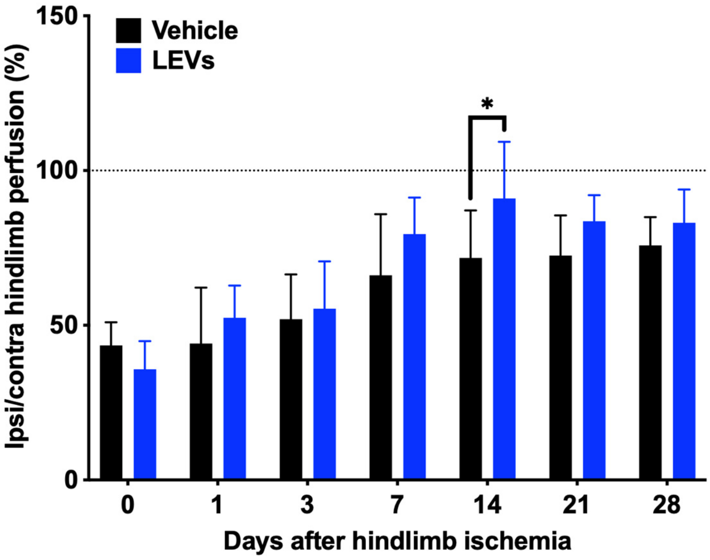

Figure S3. Quantitative analysis of LASER Doppler signal expressed as ischemic-to-contralateral muscle ratio (%, mean ± sd) from day 0 to day 28 in vehicle- (n = 10, black bars) or LEV-treated mice (n = 10, blue bars) (*P < 0.05).
